# CpG methylation signature defines human temporal lobe epilepsy and predicts drug‐resistant

**DOI:** 10.1111/cns.13394

**Published:** 2020-06-10

**Authors:** Wenbiao Xiao, Chaorong Liu, Kuo Zhong, Shangwei Ning, Rui Hou, Na Deng, Yuchen Xu, Zhaohui Luo, Yujiao Fu, Yi Zeng, Bo Xiao, Hongyu Long, Lili Long

**Affiliations:** ^1^ Department of Neurology Xiangya Hospital Central South University Changsha China; ^2^ Tsinghua‐Berkeley Shenzhen Institute Tsinghua University Shenzhen China; ^3^ College of Bioinformatics Science and Technology Harbin Medical University Harbin China; ^4^ Shanghai Biotechnology Corporation Shanghai China; ^5^ Department of Geriatrics Second Xiangya Hospital Central South University Changsha China

**Keywords:** biomarker, DNA methylation, machine learning, nomogram, temporal lobe epilepsy

## Abstract

**Aims:**

Temporal lobe epilepsy (TLE) is the most common focal epilepsy syndrome in adults and frequently develops drug resistance. Studies have investigated the value of peripheral DNA methylation signature as molecular biomarker for diagnosis or prognosis. We aimed to explore methylation biomarkers for TLE diagnosis and pharmacoresistance prediction.

**Methods:**

We initially conducted genome‐wide DNA methylation profiling in TLE patients, and then selected candidate CpGs in training cohort and validated in another independent cohort by employing machine learning algorithms. Furthermore, nomogram comprising DNA methylation and clinicopathological data was generated to predict the drug response in the entire patient cohort. Lastly, bioinformatics analysis for CpG‐associated genes was performed using Ingenuity Pathway Analysis.

**Results:**

After screening and validation, eight CpGs were identified for diagnostic biomarker with an area under the curve (AUC) of 0.81 and six CpGs for drug‐resistant prediction biomarker with an AUC of 0.79. The nomogram for drug‐resistant prediction comprised methylation risk score, disease course, seizure frequency, and hippocampal sclerosis, with AUC as high as 0.96. Bioinformatics analysis indicated drug response–related CpGs corresponding genes closely related to DNA methylation.

**Conclusions:**

This study demonstrates the ability to use peripheral DNA methylation signature as molecular biomarker for epilepsy diagnosis and drug‐resistant prediction.

## 1 INTRODUCTION

Temporal lobe epilepsy (TLE) is the most common focal epilepsy syndrome in adults and frequently develops drug resistance,^1^ requiring surgical treatment which offers a comparatively favorable prognosis.^2^, ^3^ Moreover, cognitive impairment and psychiatric comorbidities including depression and anxiety disorders, together with the long‐term actual seizures and accompanying drug usage, often result in severe effects on the quality of life and individual health.^4^, ^5^

At present, the diagnosis of epilepsy mainly depends on clinical manifestation, neuroimaging, and electroencephalogram (EEG). These methods are not only expensive and time‐consuming, but also require professional equipment and trained specialists that are not accessible to many patients, which result in delayed diagnosis or misdiagnosis to some extent.^6^, ^7^ Furthermore, drug‐response prediction is mainly based on subjective clinical features by experience and has not come to a conclude.^4^, ^8^, ^9^ Earlier identification of drug‐resistant patients makes it possible to benefit from epilepsy surgery. Thus, biomarkers for assisting the current diagnosis and predicting the treatment outcome are in urgent need. Preliminary attempts have been made in circulating molecules biomarkers of epilepsy, including inflammatory cytokines, S100 calcium‐binding protein B(S100B), and matrix metallopeptidase 9(MMP9), and recently miRNA.^10^, ^11^, ^12^, ^13^, ^14^ However, the limitations of these studies mainly related to small sample size and lack of validation, as well as heterogeneity of epilepsy that prevent the clinical value of these biomarkers.^15^

DNA methylation, the best‐studied epigenetic mechanism, refers to the covalent attachment of methyl groups to the cytosine residues (mainly confined in CpG sites) mediated by DNA methyltransferase (DNMT).^16^, ^17^ It is mostly stable throughout the genome and is associated with transcriptional activation/repression.^18^, ^19^ Aberrant DNA methylation implicated in underlying epileptogenesis and progression mechanisms of epilepsy has gained considerable attention. Altered expression of DNMTs and methylation changes in individual candidate genes (ie, *RELN*) have been found in TLE patients.^20^, ^21^, ^22^, ^23^ Several genome‐wide studies using epileptic brain tissue have identified differential methylation events occurred in genes associated with inflammation, neuronal development, etc^24^, ^25^, ^26^, ^27^ Moreover, our previous research reported that dysregulated methylation implicated in both protein‐encoding genes and noncoding RNA genes in peripheral blood DNA from TLE patients.^28^, ^29^

A substantial number of studies have investigated the value of peripheral DNA methylation signature as molecular biomarker for diagnosis or prognosis, especially in cancer research.^15^, ^30^, ^31^, ^32^ The prognostic value of O^6^‐methylguanine‐DNA‐methyltransferase (*MGMT*) promoter methylation in glioblastoma and methylated SEPTIN 9(*SEPT9*) in plasma for detection of asymptomatic colorectal cancer is well‐known paradigms,^33^, ^34^, ^35^ which have been included in clinical guidelines and translated into the commercially available clinical test.^15^ In addition, there was a trend that researchers favored combinatorial biomarkers of multiple CpG signature.^36^, ^37^, ^38^, ^39^ DNA methylation–based biomarkers present advantages with regard to clinical application: presence in various biofluids, more stable than other biological materials (such as RNA or protein), easy detection by well‐established methodologies, and cell‐type specificity.^15^, ^31^ However, to date, methylation biomarkers for TLE diagnosis and pharmacoresistance prediction have not been explored.

In this study, we aimed to identify and validate disease‐related and drug response–related CpGs in TLE. We initially conducted genome‐wide DNA methylation profiling in TLE patients; then, we selected candidate CpGs in training cohort and validated those CpGs in another independent cohort by employing machine learning algorithms. Furthermore, a nomogram comprising DNA methylation and clinicopathological data was generated to predict the drug response in the entire patient cohort. Lastly, mechanistic links were pursued for all biomarker CpGs corresponding genes by bioinformatics analysis.

## 2 MATERIALS AND METHODS

### 2.1 Patient cohorts

The study was carried out on a cohort of 78 patients with TLE and 78 sex‐ and age‐matched healthy controls, from the Department of Neurology at Xiangya Hospital. And all patients went through comprehensive medical history, physical examination, cranial magnetic resonance imaging (MRI) scans, and EEG. Inclusion criteria of TLE and drug‐resistant epilepsy were accorded to our previous research.^28^ Written informed consent was obtained from all enrolled participants. Study was conducted in accordance with the guideline for the research involving human and approved by the Ethics Committee of Central South University, Xiangya School of Medicine and the affiliated Xiangya Hospital (201303120). The data were divided into two sets: in the training cohort, 30 TLE patients were analyzed; in the validation phase, candidate CpGs were validated in another independent cohort (n = 48).

### 2.2 DNA methylation quality control and processing

Whole blood DNA extraction and quality control were constructed as in our previous study.^28^ The discovery and training samples were run on the Illumina Infinium HumanMethylation450 BeadChip Kit (450K array). The validation samples were run on the Illumina Infinium HumanMethylationEPIC BeadChip (850K array). The samples DNA underwent bisulfite treatment using the EZ‐96 DNA Methylation kit (Zymo Research Corporation, Irvine, CA, USA) and hybridized to arrays according to Illumina recommended protocols. All samples passed the Illumina quality control. Methylation at individual CpG was reported as a methylation β‐value, ranging continuously from 0 (unmethylated) to 1 (completely methylated). The minfi R package (Version 1.18.1) was used to retrieve raw data of 450K and 850K array. Initially, we excluded probes located on the sex chromosome and null probes. We also removed the failed probes with a detection *P*‐value > .05 in more than 5% samples. The probes with single‐nucleotide polymorphisms of MAF > 5% within 10 bp of the CpG sites were also rejected. We next performed Subset‐quantile Within Array Normalization (SWAN) methods for normalization.^40^ The probes of 450K assay are expected to perform similarly on data from the 850K array. In this study, we removed the set of CpG sites that were not included in the 850K array.

### 2.3 Building a diagnostic model

We included three phases to identify and validate disease‐related CpGs signature for patients with TLE. In the discovery phase, the logical regression test was performed to obtain differentially methylated CpG sites (DMCs) between 30 TLE and 30 normal control samples, with a threshold value of .001 for *P*‐value was used subsequently for filtration. The 237 candidate CpGs were analyzed by Least Absolute Shrinkage and Selection Operator (LASSO) methods. The CpGs were then ranked by the regression parameters. In the training phase, to further shrink the marker numbers to a reasonable range, support vector machine (SVM) algorithm was used for different number of CpGs. As a result, eight CpGs with the highest prediction accuracy were confirmed. SVM algorithms were tuned by 5‐fold internal cross‐validation, which implies optimal determination of parameters of the SVM algorithm. In the validation phase, the parameters of the SVM model from the training cohort were used to an independent cohort of 96 samples (48 TLE and 48 normal controls) for validating the diagnostic performance of the model.

### 2.4 Building a predictive model for drug response

In the discovery phase, the t test was performed to identify DMCs between 10 drug‐resistant and 20 drug‐responsive samples, with a threshold value of .005 for *P*‐value. After 99 DMCs were obtained, we used SVM‐Recursive Feature Elimination (SVM‐RFE) to select candidate CpG sites. In the training phase, logistic regression was used to further narrow CpGs. Six CpGs with the highest prediction accuracy were identified, with parameter tuning conducted by 5‐fold cross‐validation. A risk score was calculated for each patient using a formula derived from the methylation levels of these 6 CpGs weighted by their regression coefficient. Validation analyses were performed in another cohort (17 drug‐resistant and 13 drug‐responsive samples). In addition, a nomogram comprising integrated DNA methylation risk score and clinicopathological data was generated to predict the drug response. The performance of the nomogram was explored graphically by calibration plots.

### 2.5 Bioinformatics analysis

Pathway analysis for CpG‐associated genes was performed using Ingenuity Pathway Analysis (IPA; http://www.ingenuity.com/). For the purposes of this study, the canonical pathway and diseases functions analysis available in IPA were applied, which resulted in the inclusion of CpG corresponding genes and the other identified genes interacting with in the analysis. The Fisher's exact test was applied to measure the significance of the association between genes mapped by IPA and the canonical pathway.

### 2.6 Bisulfite pyrosequencing of selected DNA methylation loci

Bisulfite pyrosequencing is well‐established technique that used for quantitative methylation analysis of genomic regions in single‐nucleotide resolution.^41^ We selected 4 CpG loci (cg25838818, cg27564766, cg11954680, and cg26119877) for assay cross‐validation by bisulfite pyrosequencing. Blood DNA samples from 10 TLE patients and 10 healthy control cases or 10 drug‐responsive TLE cases and 10 drug‐resistant TLE cases were bisulfite converted, followed by PCR amplification of the relevant regions using the PyroMark PCR kit (Qiagen, CA, USA) according manufacturer's instruction. Nucleotide probes with biotinylated version can be detected by streptavidin sepharose, as listed below: (a) cg25838818: GTAGTTGAGGGTTAGGAAAGATGTG (F), ATACAAATACCAACTCCCTCTAATTCAT (R) and GTGAAAAATTTTAGTTGGTG (S).

(b) cg27564766: GGAGGGATAGGGGTTGTTT (F), CCAACCAACCACCTCATC (R) and TTGTGGTGGTTTATAGG (S).

(c) cg11954680: ATTAGAATTAAGAGTGATTTAGGAAGTG (F), AAAAAAAAATTTTCCTATTTCACCTTCTA (R) and GTGATTTAGGAAGTGGTTAA (S).

(d) cg26119877: AAGATTGGGTGGTTTATAAGAAAG (F), CCACAAATAAAACACATTTTACTATAACAC (R) and GTTGTTTGGGATTAGTTG (S).

Pyrosequencing assay, purification, and subsequent processing of the biotinylated single‐stranded DNA were carried out according to the manufacturer's recommendations.

### 2.7 Statistical analysis

In the comparative analysis of clinical characteristics (SPSS18.0), measurement data (age, disease course, and seizure frequency) were subject to K–S test following by statistically analyzing with Student's *t* test or nonparametric test, and enumeration data (HS, aura, and SGS) were assessed using chi‐square test, with *P*‐value < .05 considered statistically significant. For the current research, scikit‐learn (Version: 0.20) was used to perform the LASSO, SVMs, RFE, and logistic regression. The predictability of the model was evaluated by the area under the receiver operating characteristic (ROC) curve (AUC). The Youden's index was defined for all points of a ROC curve, the maximum value of which may be used as a criterion for screening the optimum cutoff point. The nomogram was constructed using the rms package in R (Version: 3.5.1). All statistical tests were two‐sided.

## 3 RESULTS

### 3.1 Characteristics of individuals

A total of 156 participants (including 10 patients with drug‐resistant epilepsy and 20 patients with drug‐responsive epilepsy and 30 healthy controls in discovery and training phase, 17 drug‐resistant, 13 drug‐responsive patients and 18 unclassified patients and 48 healthy controls in validation phase) were recruited to our study. No significant differences of age and gender were found in the training and validation set. The duration of seizures in patients with drug‐resistant epilepsy was significantly longer than that in patients with drug‐responsive epilepsy (*P* = .01 in training phases and *P* = .04 in validation phases). The pathology of hippocampal sclerosis (HS) in patients with drug‐resistant epilepsy was significantly more than that in patients with drug‐responsive epilepsy (*P* = .002 in training phases and *P* = .026 in validation phases). Seizure frequency, the existence of aura, and secondarily generalized seizure were not related to drug response of TLE patients. The detailed clinical characteristics of participants were listed in Table 1.

**Table 1:** Clinical participants of individuals

	Training set				Validation set			
	Drug‐responsive	Drug‐resistant	*P*‐value	Control	Drug‐responsive	Drug‐resistant	*P*‐value	Control
No.	20	10		30	13	17		48
Age, mean ± SD (y)	28.6 ± 10.9	31.4 ± 16.5	.65	31.3 ± 10.3	18.8 ± 10.2	31.6 ± 11.0	.32	31.5 ± 10.3
Female: male	7:13	5:5	.46	12:18	8:5	9:8	.72	25:23
Disease course, mean(range) (y)	5 (1‐24)	13 (4‐29)	.01	NA	7 (1‐13)	12 (1‐26)	.04	NA
Seizure frequency, mean(range) (/month)	3 (0.1‐120)	1 (0.3‐90)	.62	NA	2 (0.01‐75)	7.5 (1‐300)	.28	NA
HS	2 (10%)	7 (70%)	.002	NA	2 (15%)	10 (59%)	.026	NA
Aura	13 (65%)	5 (50%)	.46	NA	7 (54%)	10 (59%)	1.0	NA
SGS	12 (60%)	5 (50%)	.71	NA	10 (77%)	14 (82%)	1.0	NA

Abbreviations: HS, hippocampal sclerosis; NA, not applicable; SD, standard deviation; SGS, secondarily generalized seizure.

### 3.2 Diagnostic model for TLE

**FIGURE 1:**
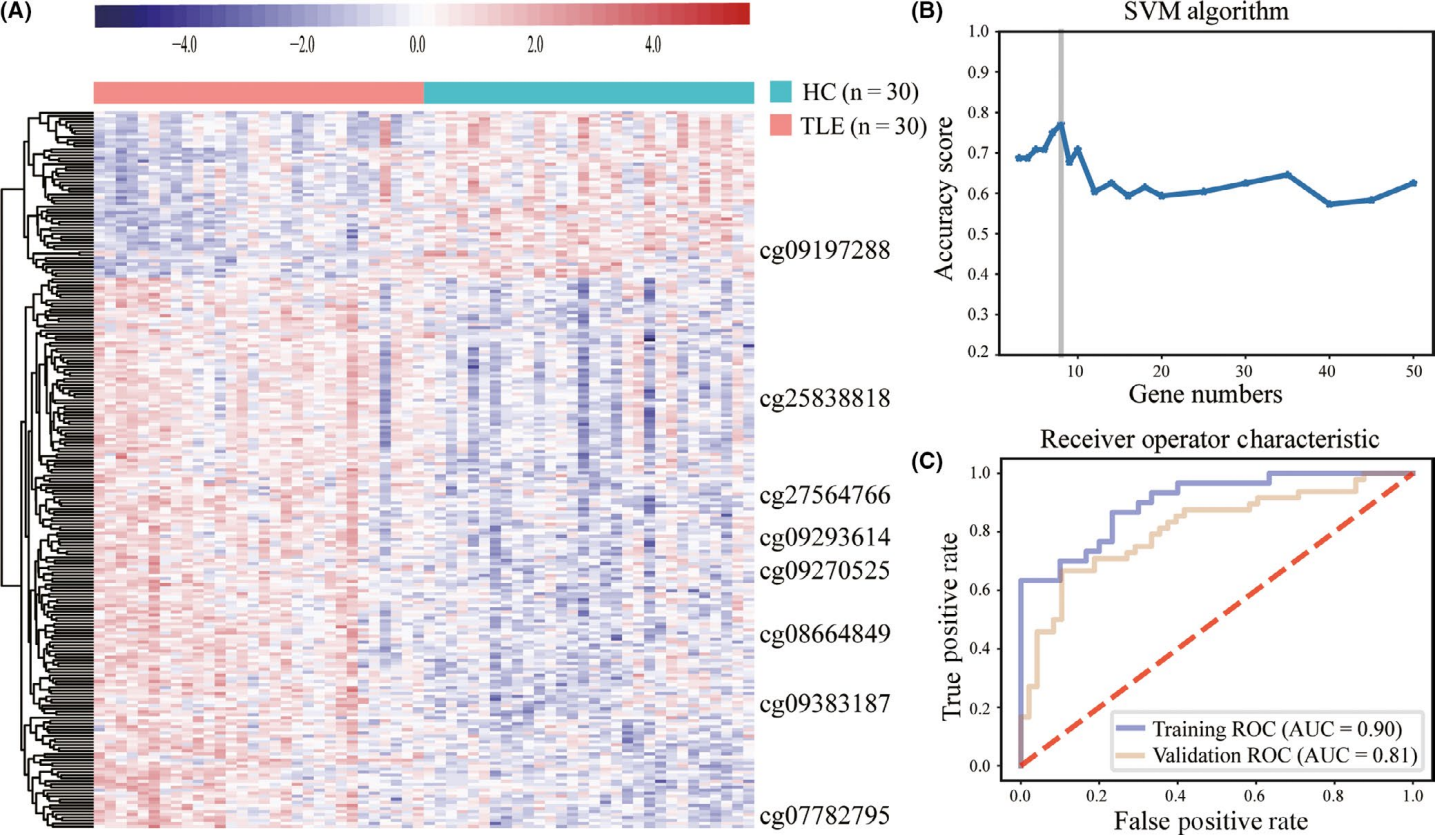
Screening and validation of disease‐related CpGs. A, Cluster analysis of 237 DMCs associated with TLE at *P* < .001 by logistic regression in the training cohort. B, SVM algorithms in the training cohort. C, Receiver operator characteristic curve of 8 significant CpGs prediction of TLE patients or healthy controls. The area under the ROC curve in training cohort was 0.90 and 0.81 for validation cohort

To identify the TLE‐associated CpGs, we first studied the global methylation profiles in the DNA of whole peripheral blood obtained from 30 TLE patients and 30 healthy controls. Epigenome‐wide association identified 237 DMCs associated with TLE at *P* < .001 by logistic regression (Figure 1A, Table S1).

LASSO algorithm was used to select the most significant CpGs from 237 candidate CpGs. We then used SVM algorithm to further narrow down the marker numbers. As a result, eight CpGs were identified (cg25838818, cg27564766, cg07782795, cg09383187, cg09293614, cg09270525, cg09197288, and cg08664849), corresponding to *SULT1C2*, *TP73*, *BAIAP2*, *CLIP2*, *MUM1*, *PTPRN2*, *IFI27L1*, and *TBC1D24*, respectively (Figure 1B, Table S2). Subsequent validation, using a separate validation cohort (n = 96), yielded a sensitivity of 71%, a specificity of 73%, and an accuracy of 77%, with an AUC of 0.81 to detect TLE (Figure 1C, Table S3).

### 3.3 Prediction model for drug response

**FIGURE 2:**
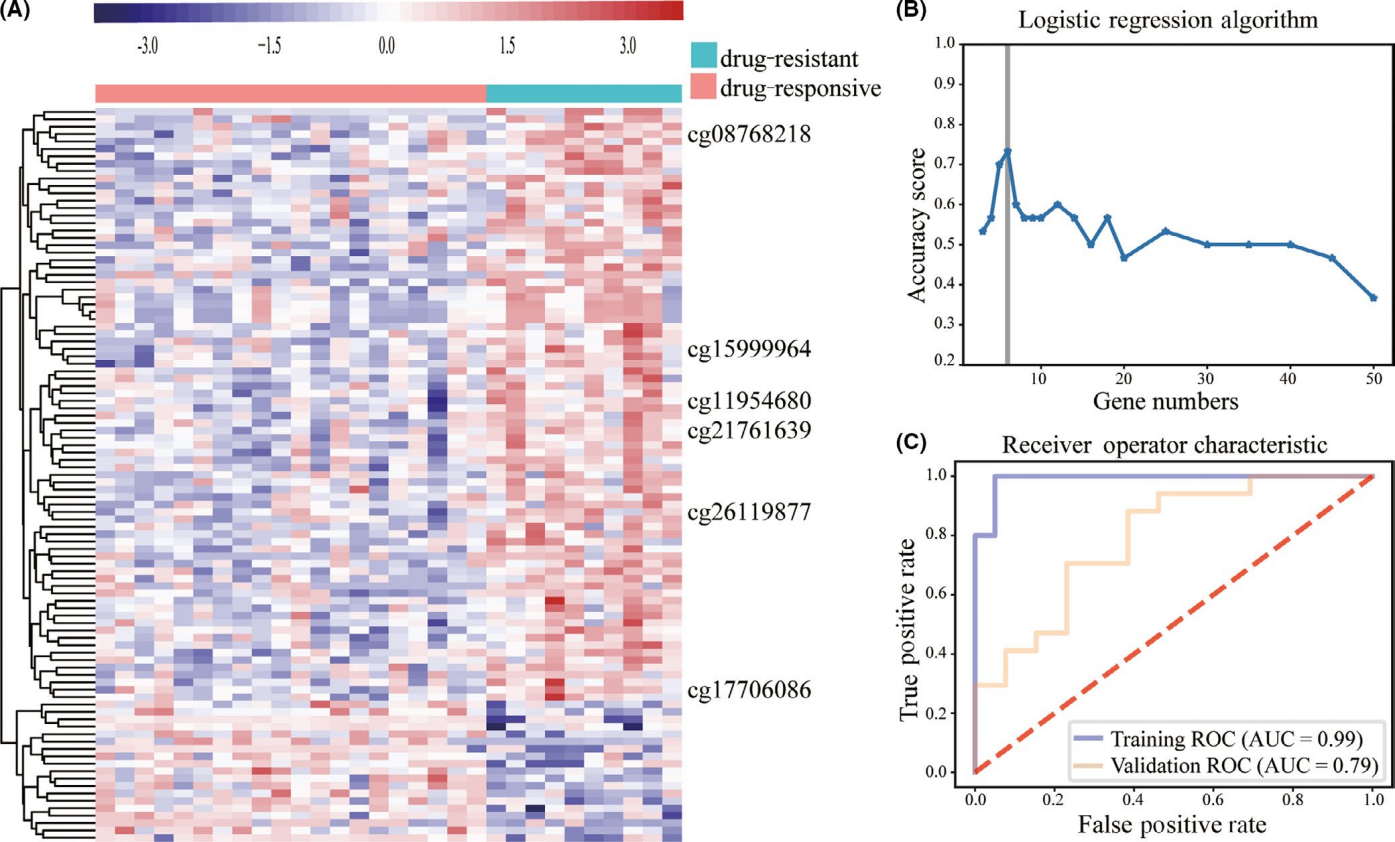
Screening and validation of drug response–related CpGs. A, Cluster analysis of 99 DMCs associated with TLE drug response at *P* < .005 by t test in the training cohort. B, Logistic regression algorithms in the training cohort. C, Receiver operator characteristic curve of 6 significant CpGs prediction of TLE patients drug‐responsive or drug‐resistant. The area under the ROC curve in training cohort was 0.99 and 0.79 for validation cohort

T test was used to analyze the DMCs between 10 drug‐resistant and 20 drug‐responsive patients, which identified 99 DMCs at *P* < .005. (Figure 2A, Table S4).

99 DMCs were analyzed by SVM‐RFE algorithm to select significant CpGs. Logistic regression was used to further narrow CpGs. Six CpGs were identified (cg15999964, cg08768218, cg11954680, cg17706086, cg21761639, and cg26119877), corresponding to *ZNF608*, *DLC1*, *PCDHA*, *MEST*, and *SLC25A21*, respectively (cg21761639 has no corresponding gene) (Figure 2B; Table S2).

To better investigate the performance of CpGs signature in predicting drug response, a methylation risk score was built with the coefficients weighted by the logistic regression model in the validation cohort (17 drug‐resistant, 13 drug‐responsive). The methylation risk score was calculated as follows: risk score = 19.3 *cg15999964 − 43.5 *cg08768218 + 54.9 *cg11954680 + 26.3 *cg17706086 + 66.8 *cg21761639 + 29.9 *cg26119877 − 86.3, with a cutoff value of 0.78. Applying the model yielded a sensitivity of 77%, a specificity of 71%, and an accuracy of 73%, with an AUC of 0.79 in the validation cohort, to distinguish drug‐responsive from drug‐resistant patients (Figure 2C, Table S5).

### 3.4 Building a predictive nomogram

**FIGURE 3:**
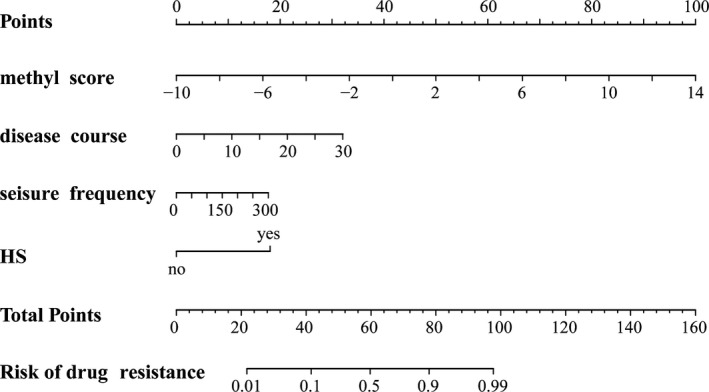
Nomogram to predict the drug response in the entire TLE patient cohort. The nomogram is used by adding up the points identified on the scale for four variables. The sum is located on the “Total points” scale, and a line is drawn downward axes to determine the risk of resistance

**FIGURE 4:**
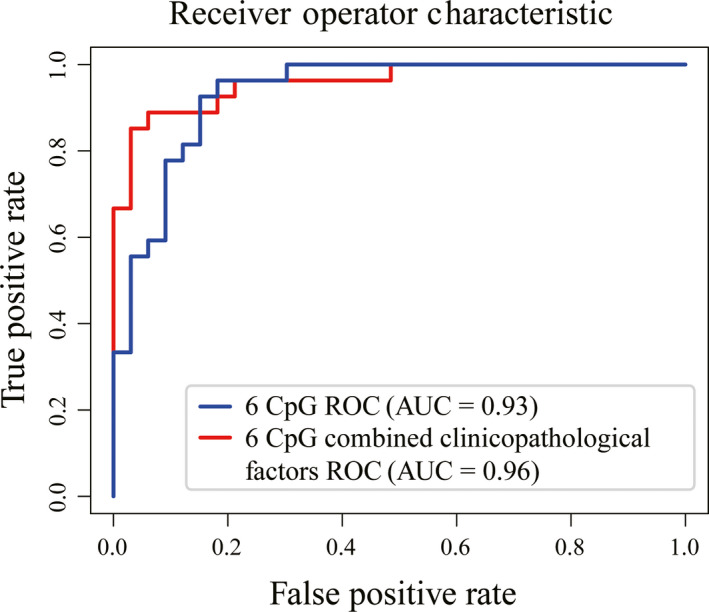
Receiver operator characteristic curve of 6 significant CpGs combined and not combined clinicopathological factors prediction of drug‐responsive or drug‐resistant in the entire TLE patient cohort

We performed the multivariate analysis of the methylation risk score and clinicopathological characteristics with drug response in the entire TLE cohort (Figure S1). The methylation risk score and HS were significantly associated with drug response. To develop a clinically applicable method that could predict drug‐resistance probability of an individual TLE patient, a nomogram was used to built a predictive model in the entire TLE cohort, taking into consideration clinicopathological factors (Figure 3). The predictors included methylation risk score, disease course, seizure frequency, and HS. Applying the model yielded a sensitivity of 94% and a specificity of 89%, with an AUC of 0.96 in the entire TLE patient cohort, to distinguish drug‐responsive from drug‐resistant patients (Figure 4). The calibration plots for drug‐response nomogram model were predicted well in the entire TLE patient cohort. (Figure S2).

### 3.5 Bioinformatics analysis

All biomarker CpGs corresponding genes were uploaded to IPA for the canonical pathway and diseases functions analysis, and network generation for defined molecular interactions. Results were visualized as networks (Figure S3, Figure S4) and ranked as diseases functions and canonical pathways involved (Table S6). The IPA analysis showed that “cell death and survival” and “cellular development” were the top‐ranked diseases functions. *DLC1*, *IFI27L1*, *TP73*, and *PTPRN2* involve in “cell death and survival” and *TBC1D24*, *BAIAP2*, *CLIP2*, and *SULT1C2* involve in “neurological disease.” Furthermore, “DNA methylation and transcriptional repression signaling” were the top‐ranked canonical pathways of 6 drug response–related CpGs corresponding genes, and *BAIAP2* involves in “axonal guidance signaling” pathway. Notably, *DLC1* is closely related *DNMT* gene (*DNMT1 and DNMT3B*) in the gene‐interaction network of 6 drug response–related CpGs corresponding genes.

### 3.6 Cross‐validation of methylation with bisulfite pyrosequencing

**FIGURE 5:**
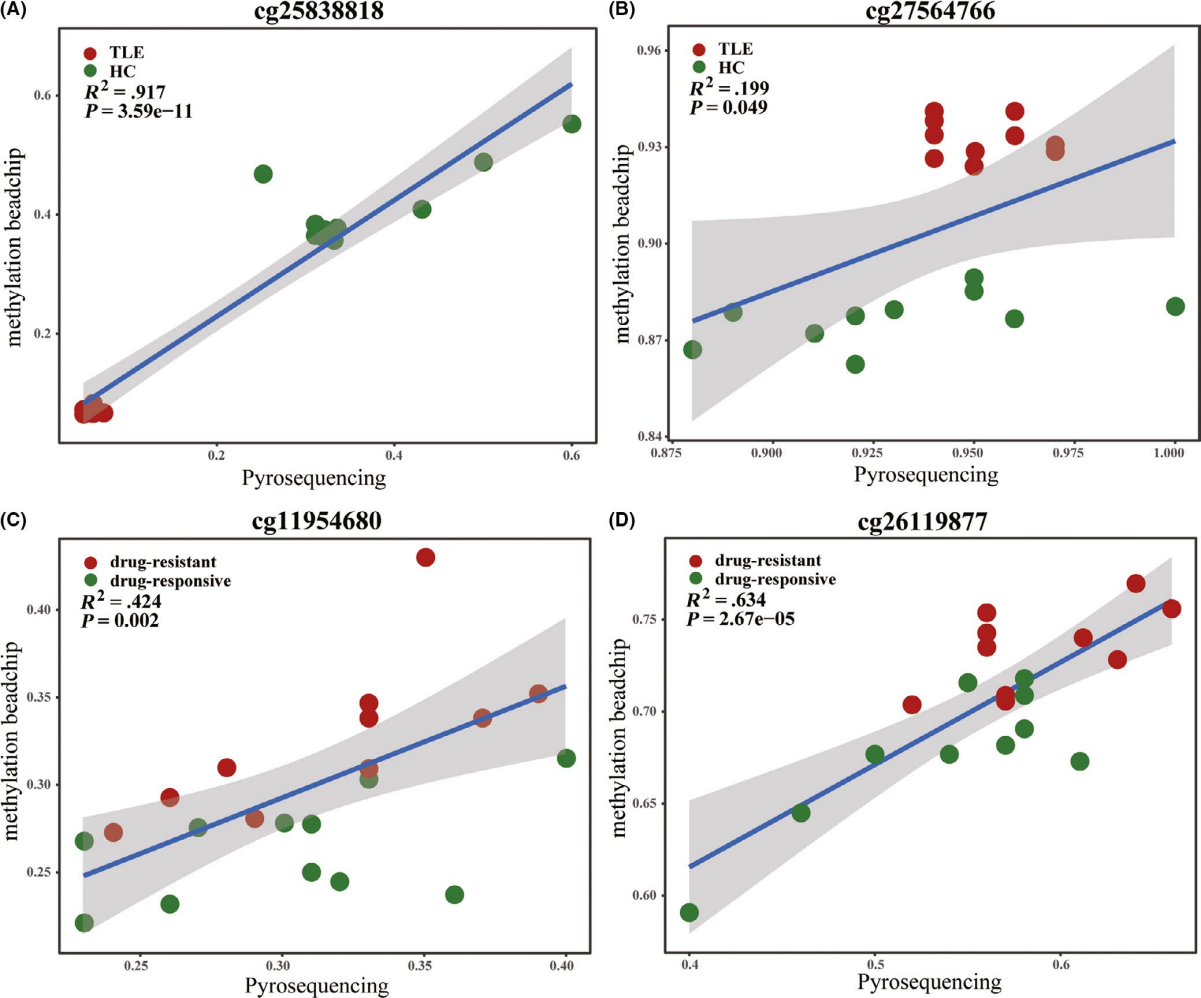
Cross‐validation of DNA methylation with the pyrosequencing. Shown are degrees of methylation of 4 CpG loci reported by methylation BeadChip (Y axis, ratio) and pyrosequencing (X axis, ratio) assays. For cg25838818 (A), cg27564766 (B), cg11954680 (C), and cg26119877 (D), the degrees of methylation detected by the two methods were positively correlated (*P* < .05) in reference to individual samples

To evaluate the accuracy of DNA methylation data from methylation beadchip, a subset of CpG loci was selected for additional methylation validation by the pyrosequencing. Blood DNA samples of TLE patients (n = 10) and controls (n = 10) were subjected to methylation detection at 2 loci (cg25838818, cg27564766), and drug‐responsive TLE (n = 10) and drug‐resistant TLE (n = 10) were subjected to methylation detection at 2 loci (cg11954680 and cg26119877). Pyrosequencing revealed methylation of cg25838818, cg27564766, cg11954680, and cg26119877 was correlated with the data from beadchip array (Figure 5A‐D).

## 4 DISCUSSION

In this study, we used the methylation array to screen differential CpGs and selected significant CpGs by applying machine learning algorithms in the training cohort. Subsequently, we validated the candidate CpGs and built the methylation‐based signature in the validation cohort. Finally, a nomogram comprising integrated DNA methylation risk score and clinicopathological data was generated to predict the drug response in the entire patient cohort. Our data indicated that the methylation‐based signature could define human TLE and predict drug‐resistant. The methylation‐based biomarker may have clinical applications for individualized diagnosis and treatment outcome prediction for patients with TLE. This study introduced a methodological framework to screen and validate biomarker and demonstrates the ability to use machine learning as a potential clinical tool for epilepsy diagnosis and drug‐response prediction after more comprehensive validation.

While great efforts have been made in understanding the underlying pathogenic and drug‐resistant mechanisms of epilepsy, there are no existing treatments to prevent or disease‐modify the development.^10^ It is believed that the complex and multifactorial features of epilepsy have led to hampered progress in these areas.^10^ Recently, epigenetics as a mediator of gene‐environment interactions had appealed to growing interesting in understanding the potential role in complex diseases.^42^, ^43^ Notably, DNA methylation is attractive to explain the underlying epileptogenesis and pharmacoresistance mechanism in chronic human epilepsy.^27^, ^44^, ^45^, ^46^ Exploring peripheral DNA methylation alterations in epilepsy is considered a direction with translational significance, given that blood samples are available in most clinical settings. Therefore, this study has several strengths should be noted. First, our focus on the specific epilepsy syndrome (TLE), the comparatively homogeneous groups of patients, advantages over the study involving different epilepsy phenotypes. This study features a genome‐wide, dual‐platform approach to screen TLE biomarkers followed by interrogation of several CpGs in a cohort of samples. Moreover, we replicated these DNA methylation signatures in another independent cohort for validation, and the machine learning model was performed well.

There are additional mechanistic links between all biomarker CpGs corresponding genes and epilepsy. For example, Tre2/Bub2/Cdc16 (TBC)1 domain family member 24 (*TBC1D24*) gene is one of the more recently discovered pathogenic mutations of familial epilepsy, of which associated disorders range from severe epileptic encephalopathy to nonsyndromic hearing loss.^47^, ^48^ *TBC1D24* has been implicated in normal neural development and survival and plays an essential role in neurotransmission and presynaptic function.^49^ In sporadic mesial TLE‐HS, whole‐exome sequencing has identified nonsynonymous de novo variants in *BAIAP2* gene, of which knockout model might produce aberrant neurological phenotypes listed by Mouse Genome Informatics (MGI).^50^ Interestingly, adenosine treatment reversed the DNA hypermethylation (including *Sult1c2* gene) status in the brain of TLE model, inhibited sprouting of mossy fibers in the hippocampus, and prevented epileptogenesis.^51^ Further characterization of molecules such as *TBC1D24*, *BAIAP2*, and *SULT1C2* will provide new insights into TLE development and progression.

In addition, we noted that drug response–related CpGs corresponding genes closely related to DNA methylation, which implicated that DNA methylation play an essential role in pharmacoresistance mechanisms of epilepsy. Tumor suppressor gene deleted in liver cancer 1 (*DLC1*) is shown to induce apoptosis, frequently silenced by methylation and negative correlation with DNMT expression.^52^, ^53^ Interestingly, previous research found increased expression of DNMT1 and DNMT3A in patients with intractable TLE.^22^ Hypermethylation of gene promoters was also the predominant effect in TLE patients and rodent models as well.^24^, ^27^, ^28^, ^29^ Given the well studied of epigenetic pathomechanisms underlying drug resistance in cancer, Kobow proposed that the methylation hypothesis of pharmacoresistance could open such new avenue in the field of epilepsy.^45^

We produced a nomogram including DNA methylation risk score, disease course, seizure frequency, and HS for estimation of individualized outcomes of the drug response in TLE patients. The AUC of predictive model is as high as 0.96, which suggests that this model is promising to be applicable in clinical practice. In the previous research, some seizure‐related characteristics, including the prior number of seizures and disease course, were reported to be related with the risk of drug resistance.^4^, ^9^ Another factor strongly linked to the increased risk of intractable epilepsy is HS, which is consistent with the findings in our study. However, limited studies applied combinatorial biomarker signatures to predict drug response in epilepsy patients. A model composed of clinical variables (the presence of HS) in combination with genetic information (SNP genotypes located in 11 genes influencing drug transport and metabolism) improved predictive accuracy for medical intractability in mesial TLE.^54^ With the advent of high‐throughput technologies and the availability of multidimensional data sets, we suggest the need to combine compound molecular approaches to achieve higher predictive performance for clinical usefulness and better comprehend the knowledge about the relevant underlying pathomechanism.

The study also has certain limits and constraints that should be noted when interpreting the results. First of all, although our classification results were promising, we should point out that high dimensional data with small sample size may result in misclassifications and biased predictors. A larger set of patients can enhance the robustness of the predictive model. Second, due to the single‐center study, the results of this pilot study warrant further validation in samples from several neurological centers. Third, TLE patients participated in this study were all being treated with antiepileptic medication, and it is still unknown whether these might affect the DNA methylation status. Furthermore, other confounding factors, such as cellular composition in whole blood, should also be taken into consideration.^55^ Fourth, two different platforms of methylation data set were hired that is 450K array for training while 850K array for validation. Several studies have reported that overall correlations of matched samples running both on the 450K and 850K array were quite high (*r* > .90 for all assessed samples).^56^, ^57^ Last, longitudinal study including the patients before antiepileptic medication is recommended to warrant clinical significance of the predictive biomarkers. The biologic mechanisms of the candidate markers are still little known, and thorough in vivo and vitro experiments are also needed to future investigate.

## 5 CONCLUSIONS

For the first time, we demonstrated that DNA methylation signature could define human TLE and compound with clinicopathological factors to improve the prediction of response to drug treatment. Furthermore, this study introduced a methodological framework to screen and validate biomarker and demonstrated the ability to use machine learning as a potential clinical investigative tool. Despite the limited pathomechanism contributions, we highlight the utilization of promising biomarkers in clinical practice for decision‐making.

## CONFLICT OF INTEREST

The authors declare no conflict of interest.

## ETHICAL APPROVAL

The study was approved by the Ethics Committee of Central South University, Xiangya School of Medicine and the affiliated Xiangya Hospital (201303120). Informed consent was obtained from the parents/legal guardians of all patients.

## Supporting information

Fig S1

Fig S2

Fig S3

Fig S4

Table S1

Table S2

Table S3

Table S4

Table S5

Table S6
