# Supplementary figures and images for: CpG methylation signature defines human temporal lobe epilepsy and predicts drug‐resistant
Source: CNS Neurosci Ther. 2020 Jun 10;26(10):1021–30. doi: 10.1111/cns.13394 (PMC7539843; doi:10.1111/cns.13394)

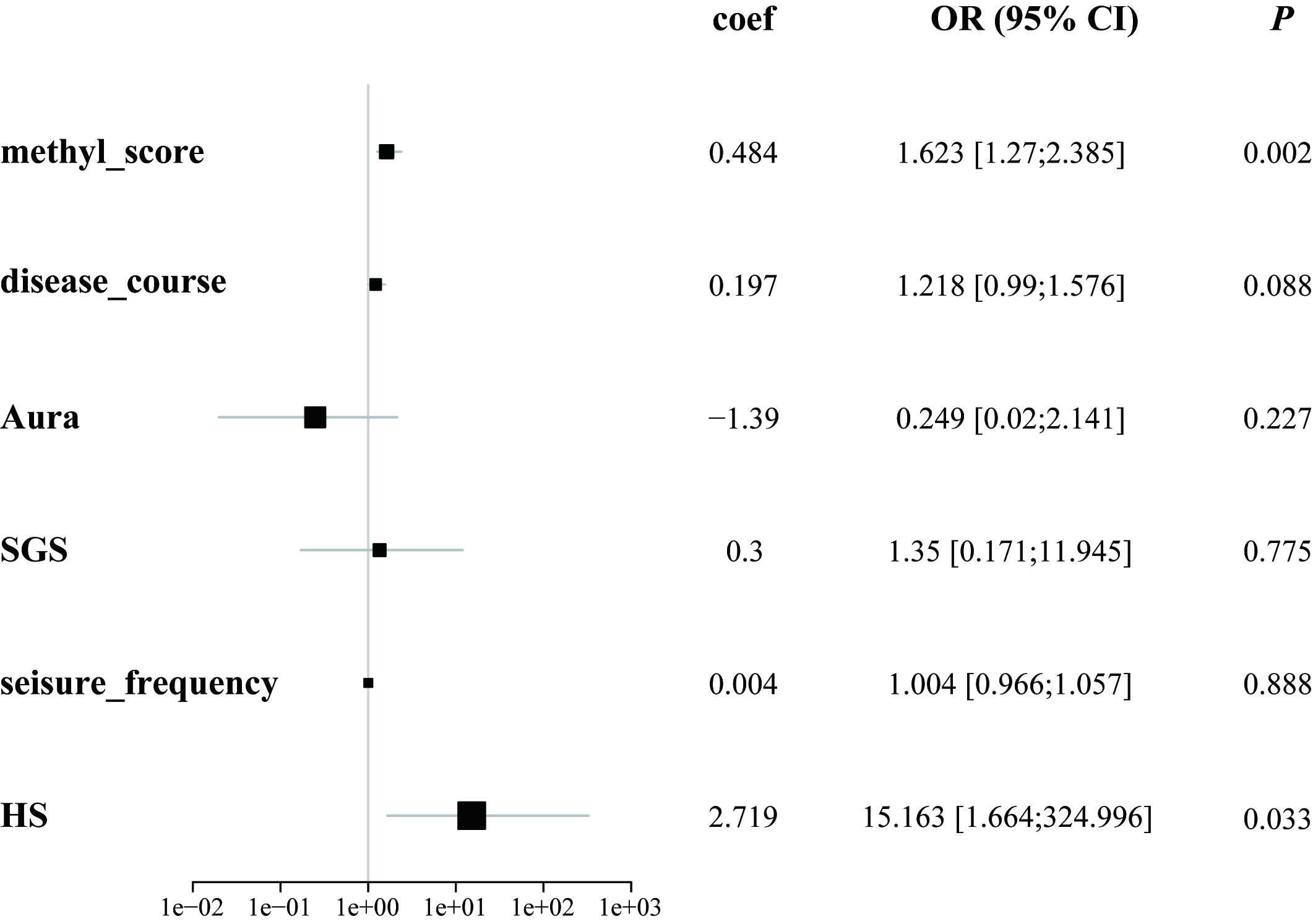

Supplement: Supplementary file 1 — Fig S1 [file CNS-26-1021-s001.tif]

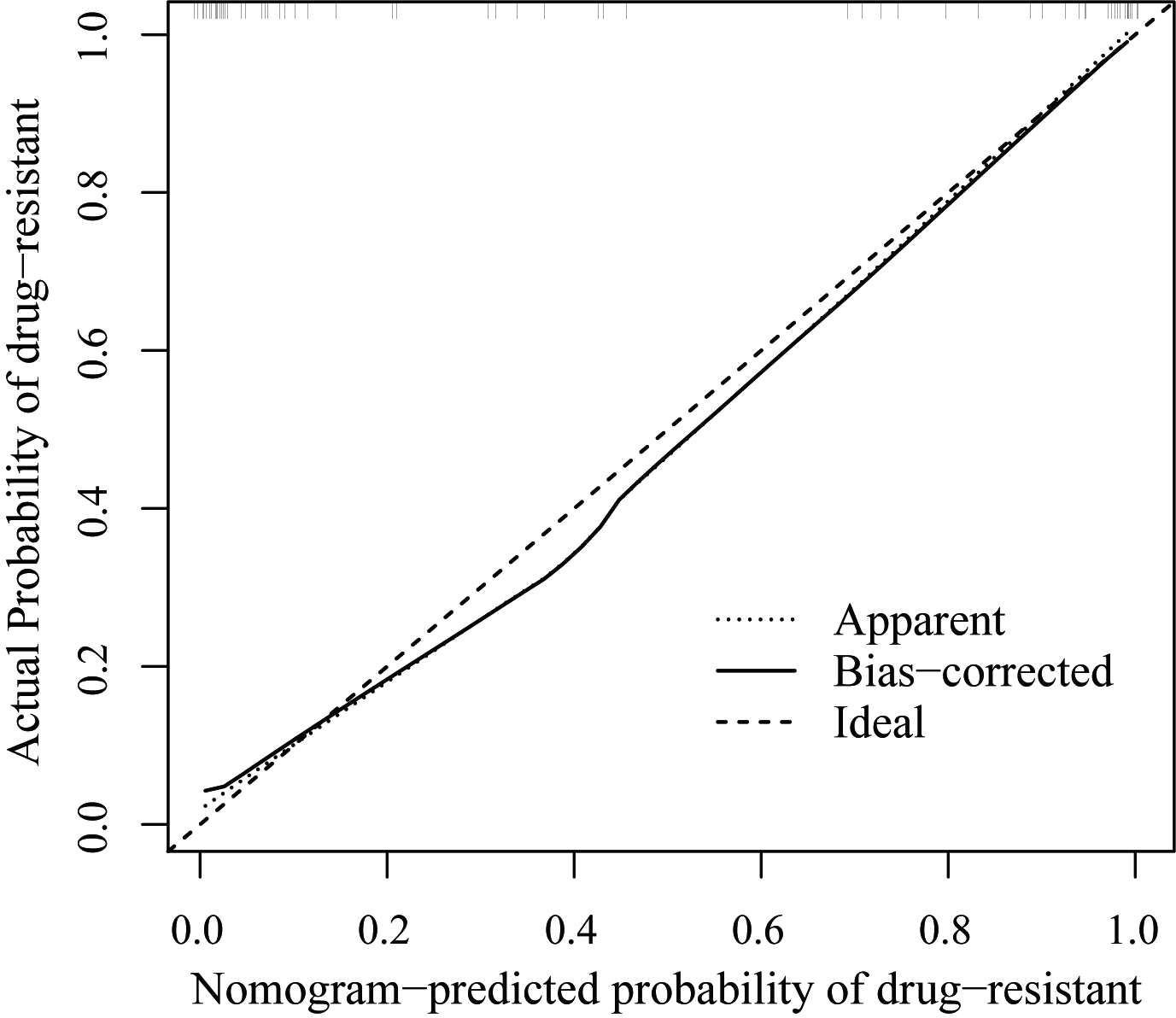

Supplement: Supplementary file 2 — Fig S2 [file CNS-26-1021-s002.tif]

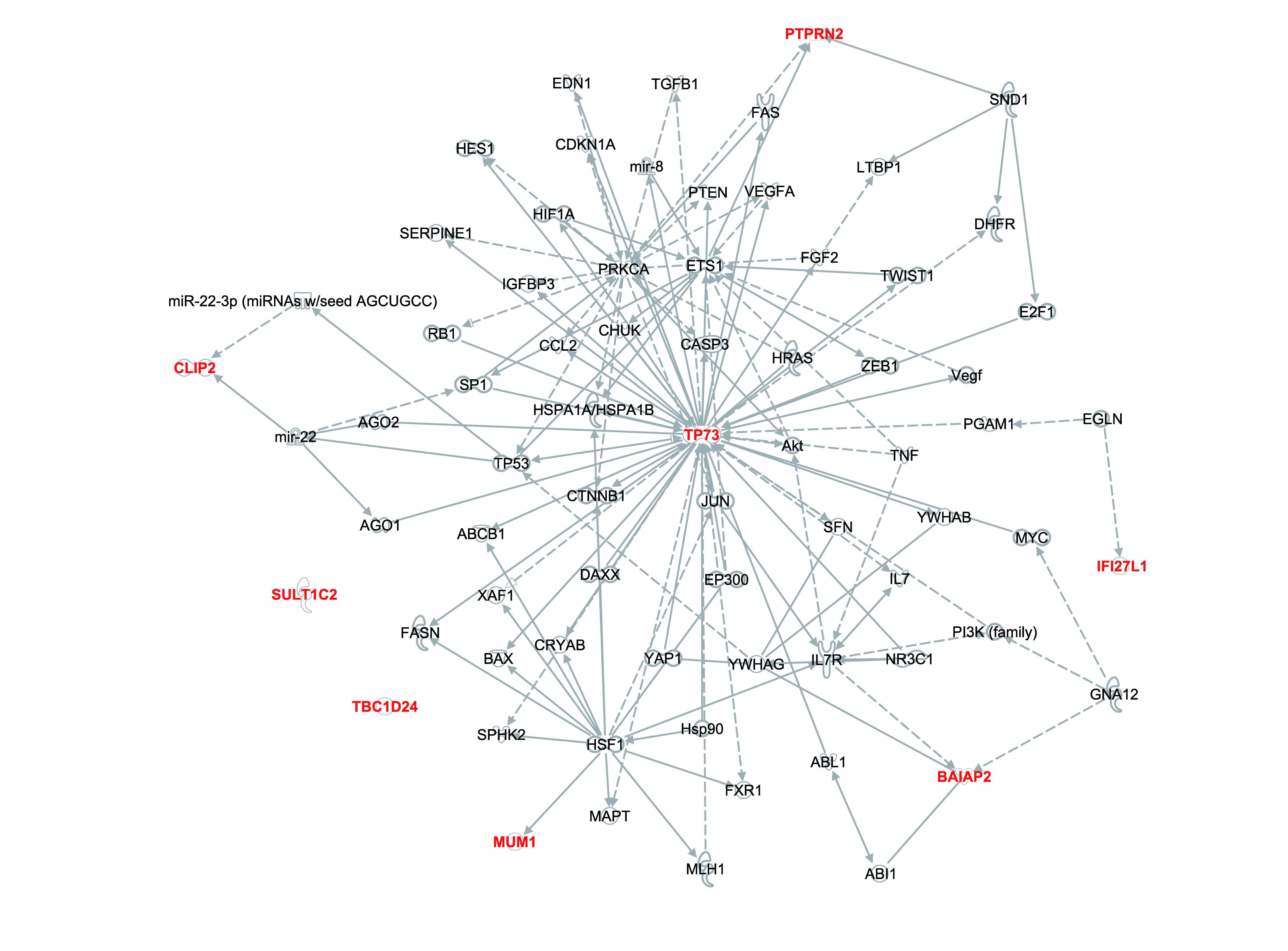

Supplement: Supplementary file 3 — Fig S3 [file CNS-26-1021-s003.tif]

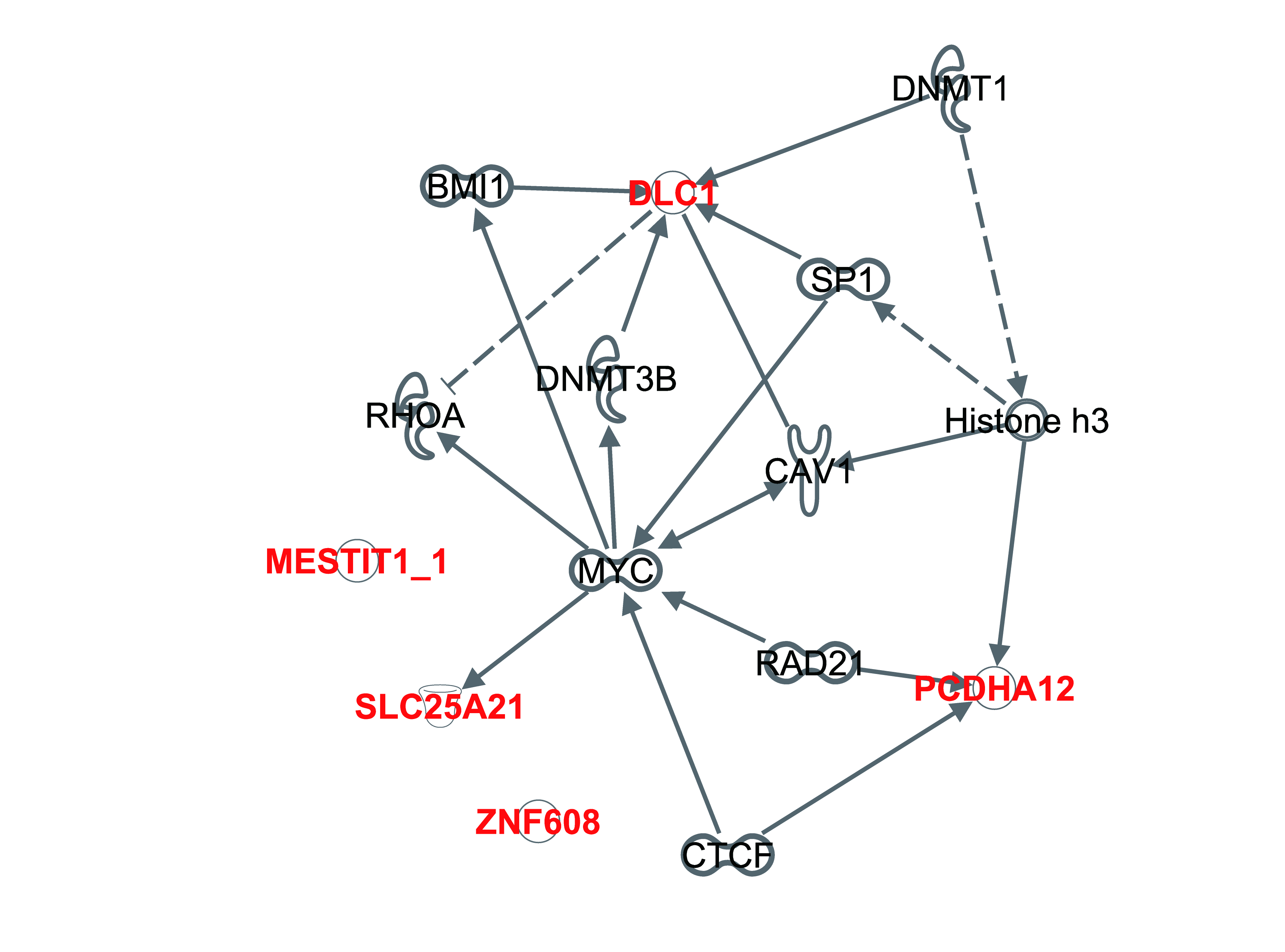

Supplement: Supplementary file 4 — Fig S4 [file CNS-26-1021-s004.tif]
